# Mind–Body Movement-Based Interventions and Periodontal Health: A Scoping Review

**DOI:** 10.3390/dj14030143

**Published:** 2026-03-05

**Authors:** Marco M. Herz, Valentin Bartha

**Affiliations:** 1 Department for Conservative Dentistry, University of Tübingen, Osianderstr. 2-8, 72076 Tuebingen, Germany; 2 Department for Conservative Dentistry, Heidelberg University, Im Neuenheimer Feld 400, 69120 Heidelberg, Germany; valentin.bartha@med.uni-heidelberg.de

**Keywords:** periodontal diseases, mind–body therapies, yoga, stress, psychological, inflammation, complementary therapies

## Abstract

**Background**: Periodontitis is a highly prevalent chronic inflammatory disease characterized by a complex host–microbe interaction and modulated by systemic regulatory pathways, including stress-related neuroendocrine and immunological mechanisms. Mind–body movement-based interventions such as yoga, tai chi, and qigong have demonstrated beneficial effects on stress and inflammation in general medicine, yet their relevance for periodontal health has not been systematically mapped. **Methods**: A scoping review was conducted in accordance with the Joanna Briggs Institute methodology and the PRISMA-ScR guidelines. Eligibility criteria included studies conducted in adult human participants examining mind–body movement-based interventions in relation to periodontal health. Sources of evidence comprised peer-reviewed studies identified through systematic searches in CINAHL, BIOSIS, Embase, PubMed/MEDLINE, the Cochrane Library, Web of Science, and LIVIVO. Data were charted using a standardized extraction form capturing key study characteristics and outcomes. Eligible studies reported clinical periodontal parameters and/or biological or psychosocial outcomes related to stress or inflammation. **Results**: Eleven studies investigating mind–body movement-based interventions and periodontal health were included. Interventions comprised yoga, pranayama, tai chi, and qigong, with study designs ranging from one randomized controlled trial to non-randomized interventional and observational studies. Most studies reported clinical periodontal parameters and/or periodontal-related biological markers, including inflammatory, oxidative, and immune markers, and several also assessed stress-related outcomes. The interventions were applied both as adjuncts to conventional periodontal therapy and as stand-alone approaches. Overall, the included studies reported short-term changes in periodontal parameters and stress-related measures that were generally directed towards associated with improvement; however, long-term periodontal outcomes were rarely assessed. **Conclusions**: Mind–body movement-based interventions, such as yoga and pranayama, have been examined in relation to periodontal health, with studies reporting periodontal clinical parameters, biological markers, and stress-related outcomes. The available evidence is heterogeneous and largely limited to short-term observations. Further methodologically rigorous studies with standardized outcome measures and longer follow-up periods are needed to better characterize the relationship between mind–body interventions and their potential adjunctive relevance in periodontal care, as current evidence does not allow conclusions regarding their routine adjunctive use.

## 1. Introduction

Over the past two decades, increasing attention has been directed toward non-pharmacological, behavioural, and lifestyle-based interventions as integral components of modern healthcare, particularly in the prevention and management of chronic diseases [1,2,3,4,5,6,7].

This growing interest reflects a broader shift toward holistic and preventive care models that address not only biomedical risk factors but also psychological, behavioural, and social determinants of health [3,8]. Chronic diseases are increasingly understood as multifactorial conditions influenced by long-term interactions between biological processes, stress exposure, health behaviours, and lifestyle patterns [9,10]. Within this context, mind–body approaches have gained prominence due to their potential to simultaneously modulate psychological well-being, physiological regulation, and behavioural engagement [1,2].

Among these approaches, mind–body concepts have gained particular relevance. These interventions integrate physical movement, breath regulation, and focused attention, aiming to enhance psychophysiological self-regulation rather than physical performance. Prominent examples include yoga, tai chi, and qigong, as well as other forms of mindful movement. Their underlying premise is that psychological, physiological, and behavioural processes are closely interconnected and can be influenced simultaneously through embodied practices [11,12].

Systematic reviews and meta-analyses in non-dental populations have demonstrated that these practices are associated with reductions in perceived stress, anxiety, and depressive symptoms, as well as favourable modulation of stress-related physiological markers, including cortisol and inflammatory mediators [13,14,15,16,17].

From a medical perspective, chronic low-grade inflammation represents a key pathophysiological mechanism linking psychological stress and lifestyle factors to a wide range of non-communicable diseases [1,2]. Stress-related dysregulation of the hypothalamic–pituitary–adrenal axis and immune system has been shown to influence inflammatory activity and disease susceptibility, providing a plausible biological pathway through which mind–body interventions may exert systemic health effects [18,19].

These mechanisms are of particular relevance to oral health and periodontology. Periodontitis is a chronic inflammatory disease driven by a dysregulated host immune response to a microbial biofilm, with established links to systemic conditions such as diabetes and cardiovascular disease [20,21]. In addition to microbial and genetic factors, psychosocial stress, depression, and anxiety have been identified as potential modifiers of periodontal disease progression and treatment response [22,23,24]. Evidence suggests that stress-related hormonal and immune alterations, including elevated cortisol levels and impaired inflammatory control, may negatively affect periodontal healing and clinical outcomes following therapy [25,26,27]. Furthermore, psychological distress can adversely influence oral health behaviours, treatment adherence, and maintenance compliance, thereby indirectly contributing to periodontal disease progression [19,24].

Given these interconnections, mind–body movement-based interventions have been proposed as potentially valuable adjuncts in periodontal care. Preliminary clinical studies have explored the effects of yoga- and breathing-based practices on periodontal parameters and salivary inflammatory markers, while a broader body of evidence supports their impact on stress reduction and immune modulation [12,15]. Within this framework, mind–body movement-based interventions may represent a relevant adjunctive approach in periodontal care. By reducing psychological stress and enhancing self-regulatory capacity, these practices could influence periodontal inflammation through both biological and behavioural mechanisms. While evidence from general medicine supports stress-reducing and potentially anti-inflammatory effects of mind–body movement practices [1,14], the dental literature remains fragmented and has not yet been systematically mapped.

Given the emerging and heterogeneous nature of the available evidence, encompassing different intervention types, study designs, and outcome measures, a scoping review approach was considered appropriate to comprehensively map the extent, characteristics, and gaps of the existing literature. From a periodontal perspective, psychosocial stress and systemic inflammation are increasingly recognized as relevant modifiers of disease expression and treatment outcomes. However, the extent to which mind–body movement-based interventions have been investigated in relation to periodontal clinical parameters and periodontal-related biological markers remains unclear. Specifically, this review addressed the following elements: adult human participants (population); mind–body movement-based interventions such as yoga, tai chi, qigong, and related practices (concept); and periodontal health outcomes within preventive and therapeutic periodontal care settings (context).

## 2. Methods

### 2.1. Study Design

This study was conducted as a scoping review to systematically map the extent, characteristics, and distribution of the available literature on mind–body movement-based interventions in relation to periodontal health and periodontal inflammation.

A scoping review methodology was chosen due to the conceptual and methodological heterogeneity of the field and the aim to identify evidence patterns and research gaps, rather than to evaluate intervention effectiveness.

The review followed the methodological framework of the Joanna Briggs Institute (JBI) and was reported in accordance with the PRISMA-ScR guidelines [28,29]. The corresponding checklist for this approach can be found in Supplementary Table S1.

### 2.2. Research Question

The research question was formulated using the PCC framework (Population–Concept–Context):

The population of interest comprised adults (≥18 years), with or without periodontal disease; the concept focused on mind–body movement-based interventions, including yoga, tai chi, qigong, and other forms of mindful movement; and the context encompassed periodontal health and inflammation, as well as stress- and inflammation-related mechanisms relevant to periodontal disease.

Primary research question:

What mind–body movement-based interventions have been investigated in relation to periodontal health or periodontal inflammation, and how is the existing evidence distributed?

#### Eligibility Criteria

Studies were eligible for inclusion if they involved adult human participants (≥18 years) and investigated mind–body movement-based interventions, such as yoga, tai chi, qigong, or related mindful movement practices, in relation to periodontal health or periodontal inflammation. Eligible studies were required to report at least one periodontal clinical parameter or a periodontal-related biological marker. All primary study designs were considered. Publications without original data, conference abstracts without accessible full text, editorials, and commentaries were excluded.

### 2.3. Search Strategy

Systematic searches were conducted in CINAHL, BIOSIS, Embase, PubMed/MEDLINE, the Cochrane Library, and Web of Science. The database LIVIVO was included to capture regional and interdisciplinary literature potentially underrepresented in biomedical databases. Database-specific search strategies combined controlled vocabulary terms (e.g., MeSH, EMTREE) and free-text terms related to mind–body movement interventions and periodontal diseases (Supplement Table S2).

The search strategy focused on peer-reviewed literature indexed in the selected databases. Gray literature sources (e.g., theses, reports, conference proceedings) were not systematically searched. No formal language restrictions were applied during the database searches. No restrictions were applied regarding study design or publication year. The final search was performed in January 2026.

### 2.4. Protocol and Registration

No formal review protocol was registered for this scoping review, which was conducted in accordance with the Joanna Briggs Institute methodology and the PRISMA-ScR guidelines.

### 2.5. Conceptual Framework and Definitions

An overview table was developed to define and categorize mind–body movement-based practices based on established literature, providing a conceptual framework for the subsequent evidence mapping. Practices were conceptually defined to provide a theoretical framework, even if some have not yet been investigated in periodontal research (Table 1).

It should be noted that the conceptual framework presented in Table 1 was developed to provide a comprehensive theoretical overview of mind–body movement practices described in the broader literature. These practices were included for conceptual clarification and contextualization, even if they have not yet been empirically investigated in periodontal research. The empirical component of this scoping review was intentionally restricted to studies reporting periodontal clinical parameters and/or periodontal-related biological markers. Consequently, only yoga, pranayama, tai chi, and qigong were included in the evidence mapping and results sections, as these were the only mind–body movement practices for which periodontal data were identified.

### 2.6. Screening and Selection Process

Study selection was conducted in accordance with the PRISMA-ScR guidelines. Title and abstract screening was performed by one reviewer, with verification by a second author. This approach was chosen in line with the exploratory nature of scoping review methodology.

Title and abstract screening were performed to assess the relevance of records to the research question addressing mind–body movement-based interventions and periodontal health. Studies were excluded at this stage if they did not report any periodontal clinical parameters (e.g., probing depth, clinical attachment level, gingival or periodontal indices) or periodontal-related biological markers (e.g., inflammatory, oxidative stress, or immunological markers), despite involving mind–body or movement-based interventions such as yoga, pranayama, Tai Chi, Qigong, or related practices. Records excluded at this stage were categorized as title/abstract excluded.

Full-text articles were retrieved and assessed for eligibility when relevance could not be determined based on the title and abstract alone. Studies were excluded after full-text review if they did not fulfill the predefined inclusion criteria, including inappropriate study population, absence of periodontal outcomes upon full-text assessment, or interventions not meeting the definition of mind–body movement-based practices. Such records were classified as did not fulfill inclusion conditions.

Records deemed non-eligible due to formal reasons only, such as duplicates, editorials, commentaries, conference abstracts without accessible full text, or publications lacking original data, were removed prior to eligibility assessment and categorized as non-eligible records removed.

**Figure 1:**
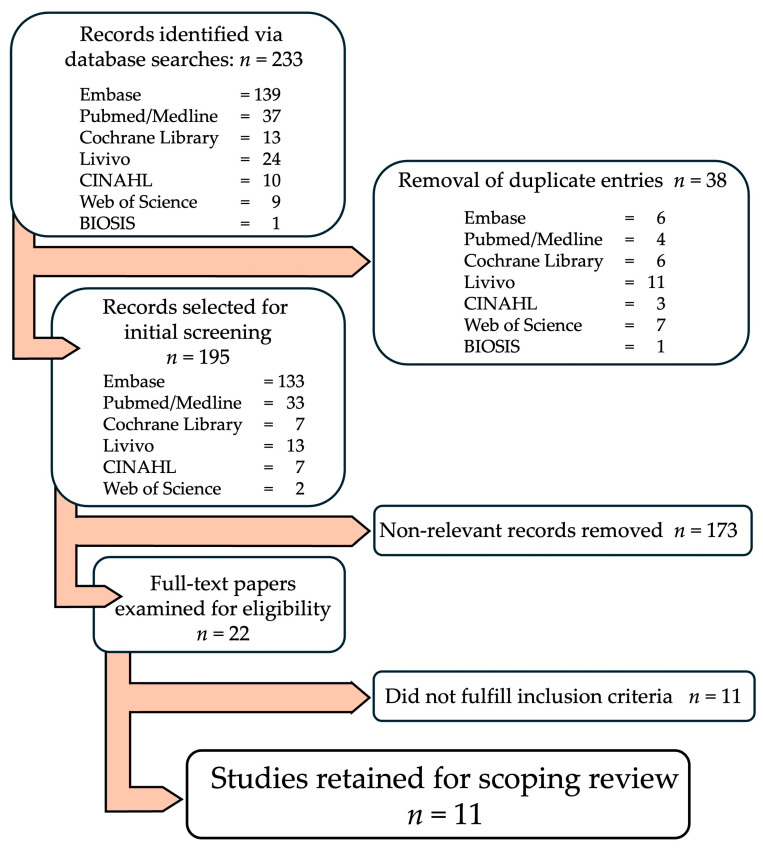
PRISMA Flowchart of the Study Selection Process.

The selection process and reasons for exclusion at each stage are summarized in the PRISMA flowchart (Figure 1).

### 2.7. Data Extraction

Data were extracted using a standardized charting form, including:

➢ Author(s), year, country
➢ Study design and sample size
➢ Intervention characteristics (type, duration, frequency)
➢ Reported outcomes (psychological, inflammatory, periodontal)
➢ Key findings
➢ Identified research gaps

Data charting Data were extracted using a standardized charting form developed by the review team. The charting form captured key study characteristics, including author(s), year, country, study design and sample size, intervention characteristics (type, duration, frequency), reported outcomes (psychological, inflammatory, and periodontal), key findings, and identified research gaps.

Data charting was performed by one reviewer and checked for accuracy and completeness by a second author. The charting form was refined iteratively during the data extraction process to ensure consistency and comprehensiveness. No additional data were sought from or confirmed with study authors.

#### Variables and Definitions

Data were charted for the following variables: bibliographic information (author, year, country); study characteristics (study design, sample size, population characteristics); intervention characteristics (type of mind–body movement practice, duration, frequency, and mode of application); and outcome measures. Outcomes were grouped into predefined domains, including periodontal clinical outcomes (e.g., probing pocket depth (PPD), clinical attachment level (CAL), bleeding on probing (BOP), plaque-related indices), periodontal-related biological markers (e.g., inflammatory, oxidative stress, or immune markers assessed in saliva or serum), and psychological or stress-related outcomes (e.g., perceived stress, anxiety, cortisol).

Due to heterogeneity in study designs and outcome reporting, outcomes were summarized descriptively and grouped by domain rather than by individual measurement instruments. No assumptions regarding effect size or clinical effectiveness were made, and qualitative weighting reflected the frequency and reported direction of findings only.

### 2.8. Critical Appraisal

No formal assessment of risk of bias or methodological quality was performed, as this is not a requirement of scoping review methodology and the aim of the review was to map the extent and nature of the available evidence rather than to assess intervention effectiveness.

### 2.9. Data Synthesis

Findings were synthesized descriptively and narratively. Studies were grouped according to intervention type, outcome domains, and relevance to periodontal health to identify evidence clusters and gaps. Charted data were organized into predefined categories and outcome domains. Due to substantial heterogeneity in study designs, interventions, and outcome measures, no quantitative synthesis was performed. Instead, data were summarized using descriptive tables and narrative synthesis, and an evidence-mapping approach was applied to illustrate the distribution and frequency of reported outcomes across intervention types. Qualitative weighting reflected the volume and reported direction of findings only and did not imply effect size or study quality.

This scoping review was conducted and reported in accordance with the PRISMA-ScR checklist.

### 2.10. Generative Artificial Intelligence

Generative artificial intelligence (GenAI) has been used in this paper to edit text (e.g., grammar, spelling, punctuation, and formatting), the standardization and optimization of tables and figures, and the harmonization of the layout (ChatGPT, version 5.2, OpenAI). The authors take full responsibility for the content.

## 3. Results

### 3.1. Study Selection Results

Database searches across all sources yielded a total of 233 records addressing mind–body movement-based interventions in relation to periodontal health and associated psychosocial and biological outcomes (Supplementary Table S1). After removal of 38 duplicates, 195 unique records were screened based on titles and abstracts, of which 173 were excluded for not meeting the predefined inclusion criteria. The full texts of 22 articles were assessed for eligibility, resulting in 11 studies being included in the final scoping review. The study selection process is illustrated in the PRISMA flow diagram (Figure 1). 

### 3.2. Characteristics of the Included Studies

The included studies were published between 2013 and 2025 and demonstrated substantial heterogeneity in terms of study design, population characteristics, and intervention protocols. These comprised one randomized controlled trial, six non-randomized interventional studies, three observational studies (one case–control study and one cross-sectional study), and one qualitative study.

Sample sizes varied considerably across studies. The smallest cohort was reported in the qualitative study involving patients with Parkinson’s disease (*n* = 15), whereas the largest cohort was observed in the cross-sectional study on yogic breathing and periodontal health (*n* = 160). Key characteristics of the included studies are summarized in Table (Table 2).

Across the included studies, mind–body movement-based interventions were implemented either as adjuncts to conventional mechanical periodontal therapy or as standalone interventions. In studies applying an adjunctive approach, all participants received standard mechanical periodontal treatment, while the intervention group additionally participated in mind–body practices. Other studies focused on psychosocial or biological outcomes without clearly reporting concurrent mechanical periodontal therapy. Importantly, the application of mind–body movement-based interventions differed with respect to their role in periodontal care. Some studies evaluated these practices as adjuncts to conventional mechanical periodontal therapy, whereas others investigated them as standalone practices without clearly defined or concurrent periodontal treatment. This heterogeneity limits direct comparison between studies and has implications for the interpretation of clinical relevance.

### 3.3. Mind–Body Movement-Based Interventions

Across the included studies, a range of mind–body movement-based interventions were identified. Yoga-based interventions were examined in four studies, followed by tai chi in two studies, qigong (Baduanjin) in one study, and breathing-focused practices such as pranayama in four studies.

Reported intervention durations ranged from 8 weeks to 6 months, while session frequencies varied between three sessions per week and daily practice. Several observational studies assessed individuals engaged in long-term yoga practice, without specifying intervention duration or weekly frequency (Table 3).

### 3.4. Reported Periodontal and Related Outcomes

The reported outcomes varied across studies and included both clinical periodontal parameters and biological or psychosocial measures. Clinical periodontal outcomes most frequently assessed were probing pocket depth (*n* = 5 studies), clinical attachment level (*n* = 2 studies), bleeding on probing (*n* = 1 study), and plaque-related indices (*n* = 2 studies).

In addition, eight studies reported biological markers related to stress, inflammation, or oxidative balance, including salivary cortisol, beta-defensin-2, nuclear factor kappa B (NF-κB), peroxisome proliferator-activated receptor gamma (PPAR-γ), superoxide dismutase (SOD), total antioxidant status (TAS), and other oxidative stress markers.

Psychosocial outcomes were assessed less frequently and included perceived stress (*n* = 2 studies) and anxiety (*n* = 1 study), while quality of life outcomes were not explicitly reported in the included studies (Table 4). Notably, psychosocial outcomes were assessed in only a small subset of studies and were typically reported as secondary outcomes rather than as primary endpoints.

### 3.5. Evidence Mapping Across Outcomes

Table 5 provides the primary descriptive evidence map of intervention types and outcome domains, while Table 6 and Table 7 offer complementary qualitative summaries of evidence distribution and reported directions of findings.

Using an evidence-mapping approach, the included studies were descriptively categorized according to intervention type and reported outcome domains. The largest proportion of studies addressed periodontal clinical parameters and periodontal-related biological markers, whereas fewer studies focused primarily on psychological or behavioural outcomes. The distribution of evidence across intervention types and outcome categories is presented in Table 5.

The evidence map indicates that yoga and pranayama were most frequently investigated in relation to periodontal clinical outcomes and periodontal-related biological markers, whereas tai chi and qigong were examined in fewer studies, with stress-related outcomes reported less consistently across all intervention types. The qualitative weighting does not reflect study quality or effect size (Table 6).

### 3.6. Summary of Evidence

Overall, the included studies predominantly reported clinical periodontal parameters and periodontal-related biological markers across yoga-, pranayama-, tai chi-, and qigong-based interventions. Stress-related outcomes were assessed less consistently and mainly as secondary measures. The distribution of evidence across intervention types and outcome domains is summarized in Table 5, Table 6 and Table 7.

## 4. Discussion

### 4.1. Principal Findings

This scoping review mapped the current literature on mind–body movement-based interventions in relation to periodontal health. Overall, the findings indicate a limited but growing body of evidence suggesting that yoga, pranayama, tai chi, and qigong may be associated with improvements in periodontal clinical parameters, periodontal-related biological markers, and stress-related outcomes [30,31,34,35]. The majority of studies focused on short-term changes in inflammation, oxidative stress, and clinical periodontal indices, while long-term periodontal stability and disease progression were rarely addressed. In relation to the research question, the review demonstrates that mind–body movement-based interventions have been explored primarily as adjunctive or supportive approaches rather than as established components of periodontal therapy. While a wide range of mind–body movement practices were conceptually defined to frame the field, empirical evidence in periodontal research remains limited to a small subset of these practices.

These findings should be interpreted as exploratory and descriptive. Due to the predominance of non-randomized study designs, small sample sizes, and short-term follow-up periods, the available evidence does not permit causal inferences regarding the effects of mind–body movement-based interventions on periodontal outcomes.

### 4.2. Interpretation in the Context of Existing Literature

The findings of this review are consistent with contemporary concepts of periodontal disease as a chronic inflammatory condition modulated by systemic, behavioural, and psychosocial factors, rather than being driven solely by microbial plaque [24,41,42,43,44]. Several included studies reported associations with improvements in probing pocket depth, gingival indices, or periodontal inflammation following yoga- or pranayama-based interventions [39,40], aligning with this host-response-centred paradigm.

A notable contribution of the included literature is the assessment of molecular and biochemical markers. Clinical trials investigating pranayama demonstrated modulation of NF-κB and PPAR-γ expression, key regulators of periodontal inflammation and tissue destruction [34,38]. However, these molecular findings are based on small, predominantly non-randomized studies and should be interpreted as hypothesis-generating rather than clinically confirmatory. These findings are supported by experimental periodontal research showing that dysregulation of NF-κB signalling contributes to exaggerated inflammatory responses and bone loss [42,45]. While these biological pathways provide plausible explanatory frameworks, the observed associations remain hypothesis-generating and should not be interpreted as evidence of causal therapeutic effects.

Similarly, tai chi and qigong interventions were associated with improvements in salivary antioxidant capacity and oxidative stress markers, alongside clinical periodontal benefits [30,32,35]. Oxidative stress is increasingly recognized as a contributor to periodontal tissue damage, particularly in aging populations and individuals with metabolic disorders [9,21]. The observed convergence of antioxidant and periodontal improvements therefore appears biologically plausible.

Stress-related outcomes further contextualize these findings. Several studies included in this review reported reductions in perceived stress or salivary cortisol among yoga or pranayama practitioners [33]. These results are consistent with a substantial body of periodontal research demonstrating associations between psychological stress, impaired immune function, and periodontal disease severity [43,46,47,48]. Activation of the hypothalamic–pituitary–adrenal axis (HPA axis) and chronic cortisol exposure have been shown to compromise periodontal wound healing and host defence [22,26,49].

Evidence from outside dentistry further supports these mechanisms. Systematic reviews have shown that yoga and tai chi can reduce systemic inflammatory markers such as C-reactive protein and interleukin-6, improve autonomic balance, and attenuate oxidative stress [50,51,52,53]. Given the bidirectional links between periodontal disease, systemic inflammation, and metabolic conditions, these systemic effects are highly relevant [21,54].

### 4.3. Implications for Periodontal Research and Clinical Practice

From a research perspective, the findings highlight the importance of integrating psychosocial and systemic dimensions into periodontal research. Mind–body interventions may influence periodontal inflammation indirectly via stress reduction, immune modulation, and improved redox balance, particularly in patients with elevated psychosocial stress or metabolic comorbidities [30,55].

From a preventive and clinical perspective, mind–body movement-based practices represent low-risk, accessible, and non-invasive strategies that may complement conventional oral hygiene, professional periodontal therapy, and supportive periodontal care. Although current evidence does not support replacing established periodontal treatments, these approaches may hold value as adjunctive elements within patient-centred, lifestyle-oriented prevention and maintenance frameworks—particularly for individuals with elevated psychosocial stress or systemic risk factors—by addressing stress-related modifiers of periodontal disease expression.

For clinical practice, these findings reinforce the relevance of interdisciplinary approaches in dentistry. Similar to their use in cardiology, rheumatology, and behavioural medicine, mind–body interventions may be cautiously integrated into dental care as adjunctive measures, particularly for stress-prone or systemically compromised patients [14,56,57,58,59,60].

Although several studies investigated mind–body movement-based interventions as adjuncts to conventional mechanical periodontal therapy, this approach was not consistently applied across the available literature. The timing, standardization, and reporting of concurrent mechanical periodontal treatment varied substantially, limiting direct comparability between studies. Moreover, a considerable proportion of studies evaluated mind–body interventions independently of active periodontal therapy, primarily focusing on psychosocial or biological markers rather than clinical periodontal outcomes. Consequently, the current evidence base does not allow definitive conclusions regarding the adjunctive clinical effectiveness of mind–body movement-based interventions in periodontal therapy.

This distinction is clinically relevant. Studies applying mind–body movement-based interventions as standalone practices cannot be interpreted as evidence for adjunctive therapeutic benefit, but rather reflect potential associations with periodontal-related biological or psychosocial outcomes. Consequently, the current evidence base does not permit conclusions regarding additive clinical effects beyond established mechanical periodontal therapy.

### 4.4. Knowledge Gaps and Directions for Future Research

Despite growing interest, several gaps remain. Most notably, long-term periodontal stability and disease progression were not adequately assessed. Chronic periodontitis is characterized by episodic progression over years, yet most studies evaluated outcomes only over weeks or months [61]. Accordingly, the current body of evidence should be interpreted as exploratory and hypothesis-generating rather than as demonstrating causal effects.

Although stress reduction is frequently proposed as a central mechanism underlying the potential relevance of mind–body movement-based interventions for periodontal health [24,43,46,47,48], only a small number of included studies assessed validated psychosocial outcomes. In most cases, stress-related measures were secondary endpoints or inferred indirectly through biological markers such as cortisol or inflammatory mediators [22,26,49]. Consequently, the proposed role of psychosocial stress reduction as a mediating pathway remains insufficiently substantiated within periodontal-specific research.

Intervention protocols varied widely with respect to duration, frequency, and intensity, limiting comparability and precluding conclusions regarding optimal intervention “dose.” Certain outcome domains, including quality of life, patient-reported outcomes, and adherence, were underrepresented. Qigong-based interventions were also sparsely investigated compared to yoga and pranayama.

Future studies should integrate validated psychosocial outcome measures alongside periodontal clinical and biological parameters. In addition, future research should prioritize randomized controlled trials with standardized periodontal case definitions, calibrated clinical measurements, mechanistic endpoints, and extended follow-up periods to better characterize potential pathways and clinical relevance [21].

### 4.5. Strengths and Limitations

Strengths of this review include a comprehensive, multi-database search strategy and adherence to the Joanna Briggs Institute methodology and PRISMA-ScR guidelines. The scoping approach enabled systematic mapping of heterogeneous evidence and identification of research gaps. Limitations include substantial heterogeneity in study design, intervention characteristics, and outcome measures, precluding quantitative synthesis. Most studies were small and non-randomized, and risk of bias was not formally assessed, which is consistent with scoping review methodology.

A key limitation of the available literature is the inconsistent application and reporting of mind–body movement-based interventions as adjuncts to mechanical periodontal therapy. Variations in treatment timing, lack of standardized periodontal protocols, and incomplete reporting of concurrent mechanical therapy limit comparability across studies and preclude conclusions regarding adjunctive clinical efficacy.

In addition, this scoping review was not prospectively registered, which should be considered a methodological limitation with respect to transparency, despite protocol registration not being a formal requirement for scoping reviews.

Furthermore, this scoping review focused on mind–body movement-based interventions in relation to periodontal health, primarily in the context of periodontitis. Studies explicitly addressing gingivitis or gingival inflammation as a primary condition were not systematically included, which should be considered when interpreting the scope of the findings. As a scoping review, this work aimed to map the breadth and characteristics of the available literature rather than to assess effectiveness or causal relationships.

Future scoping reviews may extend this framework to gingivitis as an early, reversible inflammatory condition, in which stress-related behavioural and immunological mechanisms may be particularly relevant.

## 5. Conclusions

In conclusion, the available literature suggests that mind–body movement-based interventions are increasingly investigated in relation to periodontal health with studies reporting clinical periodontal parameters, periodontal-related biological markers, and stress-related outcomes across heterogeneous study designs. However, evidence remains limited by heterogeneity and short-term designs. Rigorous long-term studies are required to clarify the role of mind–body approaches in modulating periodontal-related clinical, biological, and psychosocial domains, particularly as adjunctive strategies in periodontal prevention and care. While mind–body movement-based interventions show potential relevance in the context of periodontal health, the current evidence remains insufficient to support their routine use as adjuncts to mechanical periodontal therapy. Overall, the current body of evidence should be regarded as exploratory, reflecting associations observed primarily in non-randomized and short-term studies rather than demonstrating causal effects. Future studies employing standardized adjunctive designs and clinically relevant periodontal outcomes are required to clarify their therapeutic role.

## Figures and Tables

**Table 1:** Conceptual framework of mind–body movement practices (background literature).

Category	Method/Practice	Core Characteristics	Typical Indications
Yoga-based systems	Yoga (Hatha, Vinyasa, Yin, Restorative, Trauma-sensitive, Chair Yoga)	Integration of posture, breathing, and focused attention	Stress, anxiety, depression, sleep disturbances
East Asian movement systems	Tai Chi	Slow, continuous, meditative movement sequences	Anxiety reduction, balance, cognitive calm
	Qigong	Coordinated movement, breath regulation, mental imagery	Stress, fatigue, psychosomatic symptoms
Somatic methods	Feldenkrais Method	Exploratory, low-effort movements, sensory awareness	Stress-related tension, chronic pain
	Alexander Technique	Conscious posture and movement regulation	Stress, musculoskeletal overload
	Body–Mind Centring	Embodiment, interoceptive awareness	Emotional regulation
Mindful walking practices	Walking meditation, mindful walking	Rhythmic movement with sustained attention	Rumination, mild depressive symptoms
Dance-based mind–body forms	Authentic Movement, 5Rhythms, Biodanza	Expressive movement combined with mindful awareness	Emotional processing, stress relief
Breath–movement integration	Breath-centred movement, pranayama-based flows	Synchronization of movement and respiration	Stress, autonomic dysregulation
Therapeutic mind–body movement	Somatic movement therapy	Regulation through bodily awareness	Trauma-related psychological stress

**Table 2:** Study Characteristics: Year, Title, Journal, Design, and Sample Size (detailed).

Author	Year	Study Title	Journal	Study Design	Sample Size (*n*)
Ali Ismail et al. [30]	2025	Effect of baduanjin exercise on salivary inflammatory and oxidative markers in the elderly with metabolic syndrome and periodontal disease	Journal of Bodywork and Movement Therapies	Randomized controlled trial (RCT)	60 (30 Baduanjin/30 control)
Ananthalakshmi et al. [31]	2018	Effect of Sudarshan Kriya Pranayama on periodontal status and human salivary beta-defensin-2	Dental Research Journal (Isfahan)	Non-randomized interventional study	60 (single intervention group)
Hernández-Monjaraz et al. [32]	2013	Effect of Tai Chi on oxidative stress and periodontal disease in older adults	Free Radical Biology and Medicine	Interventional study (non-RCT)	44 (Tai Chi group; pre–post design)
Katuri et al. [33]	2016	Association of yoga practice and serum cortisol levels in chronic periodontitis patients with stress-related anxiety and depression	Journal of International Society of Preventive & Community Dentistry	Observational cross-sectional study	90 (yoga practitioners with periodontitis)
Mahendra et al. [34]	2017	Effect of Pranayama on PPAR-γ, NF-κB expressions and red complex microorganisms in patients with chronic periodontitis	Journal of Clinical and Diagnostic Research	Clinical trial (non-RCT)	40 (20 pranayama/20 control)
Mendoza-Núñez et al. [35]	2014	Tai Chi exercise increases SOD activity and total antioxidant status in saliva and is linked to an improvement of periodontal disease in the elderly	Oxidative Medicine and Cellular Longevity	Interventional study (non-RCT)	44 (22 Tai Chi/22 control)
Patanapu et al. [36]	2022	Assessment of stress and periodontal health status among individuals practicing yoga with age- and gender-matched controls	Journal of Clinical and Diagnostic Research	Case–control study	120 (60 yoga/60 control)
Raj et al. [37]	2023	Effects of yoga therapy in teaching oral hygiene practice and tooth brushing skills in patients with Parkinson’s disease	Journal of Education and Health Promotion	Qualitative study	15 (Parkinson’s disease patients)
Ramamoorthy et al. [38]	2020	Effect of Sudharshan Kriya Pranayama on salivary expression of human beta-defensin-2, PPAR-γ, and NF-κB in chronic periodontitis	Cureus	Clinical trial (non-RCT)	30 (15 pranayama/15 control)
Shukla et al. [39]	2025	Effect of yogic breathing (pranayama) on periodontal health status, salivary oxidative stress, and antioxidant levels	European Journal of Medical Research	Cross-sectional study	160 (single population sample)
Sudhanshu et al. [40]	2017	Impact of yoga on periodontal disease and stress management	International Journal of Yoga	Interventional study (non-RCT)	40 (20 yoga/20 control)

**Table 3:** Intervention duration and session frequency of included studies.

Study (Author, Year)	Intervention Type	Intervention Duration	Session Frequency
Ali Ismail et al., 2025 [30]	Qigong (Baduanjin)	12 weeks	5 sessions/week
Ananthalakshmi et al., 2018 [31]	Pranayama (Sudarshan Kriya)	90 days (~12 weeks)	Daily
Hernández-Monjaraz et al., 2013 [32]	Tai Chi	6 months	3 sessions/week
Katuri et al., 2016 [33]	Yoga	Not reported (regular practice)	Not reported
Mahendra et al., 2017 [34]	Pranayama	12 weeks	Daily
Mendoza-Núñez et al., 2014 [35]	Tai Chi	6 months	3 sessions/week
Patanapu et al., 2022 [36]	Yoga	Long-term practice (>1 year)	Regular practice (not specified)
Raj et al., 2023 [37]	Yoga therapy	8 weeks	3 sessions/week
Ramamoorthy et al., 2020 [38]	Pranayama (Sudarshan Kriya)	12 weeks	Daily
Shukla et al., 2025 [39]	Pranayama	Cross-sectional (no intervention period)	Not applicable
Sudhanshu et al., 2017 [40]	Yoga	6 months	5 sessions/week

**Table 4:** Distribution of reported outcome measures across included studies.

Outcome Domain	Specific Outcome	Number of Studies (*n*)
Clinical periodontal outcomes	Probing pocket depth (PPD)	5
	Clinical attachment level (CAL)	2
	Bleeding on probing (BOP)	1
	Plaque-related indices (PI/OHI)	2
Biological markers	Any biological marker	8
	Salivary cortisol	2
	Inflammatory signalling (NF-κB, PPAR-γ)	2
	Antioxidant/oxidative stress markers (SOD, TAS, OS)	4
Psychosocial outcomes	Perceived stress	2
	Anxiety	1
	Quality of life	0

**Table 5:** Evidence map of intervention types and outcome domains.

Intervention Type	Periodontal Clinical Outcomes	Periodontal Biological Markers	Psychological Outcomes	Behavioural/Oral Hygiene Outcomes	Total Studies
Yoga	3	2	1	1	4
Pranayama (breathing-based)	3	3	1	0	4
Tai Chi	2	2	0	0	2
Qigong (Baduanjin)	0	1	0	0	1
Total	8	8	2	1	11

Notes: Numbers indicate the number of studies reporting each outcome domain; individual studies may contribute to more than one outcome category. Outcome domain definitions: Periodontal clinical outcomes: probing pocket depth, clinical attachment level, gingival/periodontal indices, CPI. Periodontal biological markers: inflammatory markers, oxidative stress markers, immune mediators in saliva or serum. Psychological outcomes: stress, anxiety, cortisol. Behavioural/oral hygiene outcomes: tooth brushing skills, oral hygiene practices.

**Table 6:** Evidence map of mind–body movement practices and periodontal-related outcome domains.

	Outcome	Periodontal Clinical Outcomes	Periodontal Biological Markers	Stress-Related Outcomes
Practice				
Yoga		✖✖✖	✖✖	✖
Pranayama (breathing-based)		✖✖✖	✖✖✖	✖✖
Tai Chi		✖✖	✖✖	✖
Qigong (Baduanjin)		✖	✖✖	✖

Legend: ✖✖✖ = several studies, generally consistent direction of findings. ✖✖ = few studies with consistent or largely consistent findings. ✖ = single study or very limited evidence. Notes: This qualitative weighting reflects the frequency and reported direction of findings only and does not imply effectiveness, causality, or study quality. The evidence map reflects the number of included studies reporting each outcome domain. Ratings are based on the volume of studies and consistency of reported directions, not on effect size or study quality. Individual studies may contribute to more than one outcome category.

**Table 7:** Summary of reported effects across included studies.

Outcome Category	Direction of Reported Effects	Number of Studies (*n)*	Notes
Periodontal clinical parameters (PPD, CAL, CPI, GI)	Improvement reported (short-term, heterogeneous designs)	6	Mostly short-term interventions
Periodontal biological markers (inflammation, oxidative stress)	Improvement reported (short-term, heterogeneous designs)	7	Consistent direction, heterogeneous markers
Psychological outcomes (stress, anxiety)	Reduction reported	3	Often secondary outcomes
Long-term periodontal stability	Not adequately assessed	0	No long-term follow-up
No clear or mixed effects	Mixed/unclear	2-3	Mainly observational or cross-sectional designs

Notes: Numbers indicate the number of studies reporting effects in each category. Due to heterogeneity in study design, interventions, and outcome measures, no quantitative synthesis was performed.

## Data Availability

The original contributions presented in this study are included in the article and Supplementary Materials. Further inquiries can be directed to the corresponding author.

## Supplementary Materials

The following supporting information can be downloaded at: https://www.mdpi.com/article/10.3390/dj14030143/s1, Supplement Table S1: Preferred Reporting Items for Systematic reviews and Meta-Analyses extension for Scoping Reviews (PRISMA-ScR) Checklist; Supplement Table S2: Database-specific search strategies. Reference [62] is cited in the supplementary materials.

## Abbreviations

The following abbreviations are used in this manuscript:

BOP	Bleeding on probing
CAL	Clinical attachment level
CPI	Community periodontal index
MeSH	Medical Subject Headings
NF-κB	Nuclear factor kappa B
PCC	Population–Concept–Context
PPAR-γ	Peroxisome proliferator-activated receptor gamma
PPD	Periodontal pocket depth
PRISMA-ScR	Preferred Reporting Items for Systematic reviews and Meta-Analyses extension for Scoping Reviews
SOD	Superoxide dismutase
